# Additional Experiments on the Arteries of Warm-Blooded Animals; Together with a Brief Examination of Certain Arguments Which Have Been Advanced against the Doctrines Maintained by the Author of " an Experimental Inquiry, &c."

**Published:** 1819-10-01

**Authors:** 


*Additional Experiments on the Arteries of Warm-blooded Animals; together with a brief Examination of certain Arguments which have been advanced against the Doctrines maintained by the Author of "An Experimental Inquiry, &c."* By CHARLES HENRY PARRY, M. D. F. R S. Physician to the Bath General and Casualty Hospitals, and to the Puerperal Charity in that City. One Volume, Octavo, 258 Pages, with Six Plates. London, 1819.

### V.

*An Essay on the Forces which circulate the Blood; being an Examination of the Difference of the Motions of Fluids in Living and Dead Vessels.* By CHARLES BELL, F. R. S. E. Surgeon to the Middlesex Hospital, &c. One Vol. Duodecimo, 83 Pages. London, 1819.

WHEN we say that the *blood returns or is returned* (for we dare not be positive on this point) from the general venous system to the right auricle of the heart, from thence to the ventricle, from the ventricle to the lungs, from the lungs to the left side of the heart, and from thence to the general arterial system, we state almost the whole of our unquestioned knowledge relative to the circulation of this fluid. After all the experiments that have been made on the living and the dead—on the cold and the warm-blooded—with microscopes and with injections, the physiological world is as much divided on this interesting problem as in the days of Harvey. Independently of the numerous papers in periodical journals, four distinct works on the circulation of the blood, have, within these two or three years, been published, and four *different* explanations attempted of what was supposed to be settled by Harvey.* This is sufficient evidence that we have yet much to learn on the subject; and we think it behoves the different parties in this discussion to divest themselves, as much as possible, of preconceived opinions, and of that influence which great names impose, in order to be open to the arguments as well as the facts, brought forward by each other.

The readers of Dr. Parry's work on the pulse, of which

* See Mr. Kerr, Dr. Carson, Dr. Parry, and Mr. Charles Bell, on the Circulation of the Blood.

we gave an analysis in the second volume of our Monthly Series, will remember that three rams were left by the author, under experiment; one with a ligature on the left carotid, and two with both carotids tied. The result was to have been given in an Appendix; but, by an awful and afflicting dispensation of Providence, the functions of that distinguished physician's clear and vigorous mind were suddenly suspended, and the energies of a most active frame arrested, in the midst of health, and hope, and occupation! By this melancholy event, medical science has lost one of its brightest ornaments and ablest cultivators! Dr. Parry's "Elements of Pathology and Therapeutics" will stand a lasting monument of fame to the author, while, as an unfinished work, it must ever excite the deepest sorrow throughout the profession, for his premature fall!

The present Essay is intended by his son, Dr. Charles Parry, as a supplement to his father's work on the pulse, in which he "has endeavoured to give an accurate account of the experiments which were in progress," together with some others of his own, bearing on the same subject.

In the first experiment, the left carotid of the ram had been tied by a single ligature, exactly in the middle of the exposed part, on the 20th of April, 1815. On the 19th of August, 1817, this ram was destroyed, by opening the aorta; and on the 21st was injected by Mr. Norman from the innominata. The injection passed well through the right carotid, but the communication was not perfect on the left side, the artery being filled chiefly by return fluid. It was found that the vessel had separated where first tied, and that the parts had receded to a considerable extent. *Two new vessels* immediately connected the divided ends of the artery, and certain anastomosing branches assisted in carrying on the circulation. Hence it is evident that the phenomena had been similar to those exhibited in former experiments; and that the supply of blood on this side had been provided by *new* arteries growing out of the sides of the old. It was also observed, that the *right* carotid of the animal had *relatively* increased in size, which increase of calibre probably compensated for the contracted dimensions of the opposite artery, and the fewness and small size of the vessels which carried on the circulat on on the left side.

The second experiment confirmed the preceding. A portion of the carotid of a sheep had been cut out, and on dissectio 1, nine months afterwards, a congeries of vessels appeared in direct communication with each arterial stump. In the third experiment ligatures were applied, at the same instant, to both carotids of a ram, and eleven months afterwards the animal was killed and injected. The vessels on both the right and left side, above and below the place of ligature, were preternaturally large, and a number of clear and distinct tubes were found springing directly from the stump on either side. Besides these direct vessels of connection, there was in each artery a large arch of communication, which probably conveyed the blood previously to the formation of these numerous connecting vessels. Whether this arch was itself a new formation, could not be ascertained.

Such is the result of the experiments which were in progress when the elder Dr. Parry published his work on the pulse. The younger Dr Parry has had opportunities of repeating and extending these trials on the same and other animals, and details them in this place. These trials correspond with the former; but in one of them, where a large portion (an inch) of both carotid and par vagum was cut out, not only was the circulation re-established by a series of new vessels, but the vagus nerve, after having united itself with the coats of the artery at the place of the ligature, "*had extended itself, in company with the newly-formed vessels, to the other extremity of the artery.*"

In these experiments, Dr. Parry took an opportunity of verifying his father's conclusions, relative to the non-dilatation and contraction of the arteries at each systole and diastole of the heart.

Although it is of little importance to science, as Dr. Parry observes, whether these facts were first noticed by his father or not; yet the merit of having at least confirmed a principle, which was before questionable, cannot be refused him. And this, as is the case with most other discoveries, was first asserted to be *untrue*; and when the fact could no longer be denied, it was affirmed to be—*not new!* The admirers of John Hunter have found, as usual, or fancied they found, a similar discovery in his writings; but, as our author observes, "It will indeed prove either a very microscopic eye, or the facility of greatly magnifying appearances, to discover in Mr. Hunter's ingenious observations on the subject of restored circulation, any evidence of corresponding facts, or of a principle corresponding to that developed in the "Inquiry." P. 25.

With that candour, however, which is characteristic of great minds, Dr. Parry brings forward a curious document which, in some measure, deprives his father of the honour of being the *first* discoverer of this interesting fact. M. Maunoir, in his "Memoires sur L'Aneurisme," relates the reults of an experiment which he made on the right carotid of a fox, by tying two ligatures on it, and cutting them between. On examination afterwards, a vessel was found passing from one extremity of the divided vessel to the other, which he took for a *regeneration of the original trunk,* but which was evidently a new vessel.

We cannot follow Dr. Parry through his very ingenious observations on the *manner* in which Nature effects this remarkable renovation of tubes and circulation; but refer to the volume itself, from page 32 to page 54.

Among the various objections which have been raised against Dr. Parry's experiments and conclusions relative to the non-dilatation and contraction of arteries, at each systole and diastole of the heart, we shall notice the following.

1st. It is contended that arteries must dilate and contract, because their dilatation is seen and felt.

2d. That the pulse must arise from dilatation and contraction, because this alternate movement is distinguishable by sight and touch.

3d. That arteries must, under common circumstances of circulation, dilate and contract, *because* they are not "*inert tubes,*" and because they have, under certain circumstances, an action independent of the heart.

To the first objection it is sufficient to state that, in all Dr. Parry's accurate experiments on the arteries of living animals, no such alternate dilatation and contraction was ever observable by himself or any of those gentlemen who saw and felt the vessels when laid bare. Mr. John Hunter himself, indeed, long ago acknowledged that in cutting upon an artery, the nearer you came to it, the less perceptible was its motions, and when you laid it quite bare, no motion, and consequently no dilatation or contraction was visible or tangible.* The most distinguished and accurate physiologists on the continent, are now unanimous that no such alternate dilatation and contraction as has been contended for, exists in arteries, as the following quotation from the great French work, now publishing, will shew. The Editors, after stating facts analogous to those of Dr. Parry, conclude thus:

"Or, quand deux sens, *la vue et le toucher*, aussi parfaits par leur reunion, ne nous disent rien, comment oser recourir au rai-

---

* On the blood, vol. i, page 307.

sonnement, pour admettre un mouvement alternatif de dilatation et de contraction dans les arteres."

*Dict. des Sciences Med. tom. 5, p. 236.*

Spallanzani's experiments have been triumphantly brought forward on this subject, but with very little reason, as the following facts will shew. In the first place, his experiments were *microscopical,* which are proverbially liable to error. In the second place, they were principally made on the aorta of salamanders and other animals in whom that vessel participates in the structure and functions of the heart, and in fact may be considered as an accessory portion or rather elongation of that organ. As to the instances where the animals were *akerious,* and where the large vessels necessarily performed the *vicarious* office of the heart, they furnish no analogy, or argument on either side.

But after all, Spallanzani believed that the heart was the sole agent in the circulation, and that its impulse extended even to the veins.

"If the vessels, (says he) really experienced this alternation of contraction and dilatation, and an accelerated momentum, these phenomena should be evident; nevertheless, the result of allmy observations tends to establish a complete inertia in the coats of the capillary branches, and a retardation rather than an augmentation of velocity in the circulation. I have also been taught by the evidence of my senses, that the course of the blood is independent of the contraction of the artery; since, in several large animals it was only evident in the largest trunks; and in embryos we perceived no appearance of contraction or dilatation throughout the vascular system. It is easy to perceive that the circulation of the blood depends solely on the action of the heart."

Whoever judges Haller by the spirit of his general opinions with regard to arteries, rather than by detached passages, will be convinced that he entertained very great doubts as to the doctrine of dilatation and contraction being the cause of the pulse. It is well known, that he maintained the sufficiency of the heart to carry on the circulation; and believed that arterial dilatation and contraction impeded rather than accelerated the motion; he also admitted the fact of pulsation, where no alternate movement in the artery had taken place.

Even Senac admits that in cold blooded animals there is no positive evidence of alternate dilatation and contraction in the arteries, which may be compared, in his opinion, to tubes of glass. He maintains too, that in man, the dilatations cannot be seen. "Les yeux ne peuvent pas saisir cette suite successive de la dilatation dans les arteres."

Lewenhoek, the chief of microscopic observers, never saw the dilatation and contraction of arteries in the course of his multifarious experiments on the circulation of animals.

Bichat, the modern Haller, whose ample means of acquiring knowledge were most happily applied to the extension and arrangement of important principles, has taken great pains to shew that the arteries do not possess this alternate motion of contraction and dilatation, and his proofs that the pulse is not occasioned by any vital action of the arteries, will be sufficiently satisfactory to those who peruse them uninfluenced by former opinions, and the authority of names.

The opinions of Richerand and Dumas are unsupported by any direct evidence, and Portal inclines, at least, as much to loco-motion as to dilatation of the artery.

Mr. Charles Bell, in his beautiful "System of Dissections,' affirms, that the pulsation in the arteries is occasioned by the whole quantity of blood sent through them in the direction of their axis; while the philosophic and scientific Dr. Young, after examining the various powers which move the blood, concludes that the nature of the pulse, as perceptible to the touch, must obviously depend entirely on the action of the heart, since the state of the arteries can produce very little alteration in its qualities.

Upon the whole, the evidence of non-dilatation and non-contraction, in the ordinary state of the circulation, and as regards the pulse, is so preponderating, that it is hardly credible that any man can continue to support the old doctrine, who is at all open to the evidence of truth.

So far we have travelled in great unison with Dr. Charles Parry; but we are sorry that we are now forced to separate from so instructive and sensible a companion, and take a different path. In that division of the work which treats of "the power of arteries," Dr. Parry strongly advocates the cause of what is called *determination* of blood *by the heart alone*, to particular vessels, or sets of vessels, and that without any preceding change in the vessels themselves. We have long reflected on this subject, and we hold correspondence with some of the brightest ornaments of the profession, who unanimously deny the heart any such power.

"We know, says Dr. Charles Parry, that the heart can, under circumstances, occasion the distention of particular vessels, in which no previous alteration has taken place, apparently by the mere exercise of its ordinary functions, and by the simple propulsion of an excessive quantity of blood which belongs to the general system, or has accumulated from obstruction in a neighbouring series. 117.

The heart throws a quantum of blood, at or above par, into the aorta; but by what means it can direct the stream, when it arrives at the bifurcation or the illiacs, to take to the one instead of the other, *without any preceding change in either*, we are totally at a loss to conjecture. If it have this power over the direction of the blood, after the current has left the heart and primary trunks, it must be according to some law unknown to us, either in philosophy or medicine.

Dr. Parry attempts to illustrate his doctrine by the phenomenon of *blushing*, &c.

"Who, (says he,) that has nicely examined these sensations will argue with regard to the deep blush of shame or modesty, that a prior sensation was not felt by the heart, and that the heart was not the occasion of the distention of those vessels." P. 109.

Dr. Parry will hardly argue that the impression of shame, or any other emotion, is not primarily made on the brain and sentient system, and that the heart comes not to be affected secondarily. Now we see no just reason why the subcutaneous vessels of the cheek should not feel the influence of this mental emotion, as well as the heart; and that this sudden distention of the vessels of the face and neck, in blushing, is owing to the state of the nerves spread among them, we have not the smallest doubt, and we believe it is the opinion of the best physiologists of the present day. We know that friction, for instance, or any other thing that excites the nerves of a part, will be instantly followed by a *blush* of that part, without any alteration in the rhythm of the heart:—even the *imagination* will act on the nervous structure of certain organs, which need not be specified here, and the consequence will be an erythism, or as the continentalists term it, an *erection* of those vessels, with a corresponding rush of blood into their cavities. We believe that this local influence of the nerves of a part in dilating the vessels thereof, will offer a more rational explanation of what is denominated "*a determination* of blood to a part," than any supposed elective power in the heart over the blood when once emancipated from the cavities of the organ and primary trunks.

Such indeed appears to be the opinion of the elder Dr.

Parry himself, though he has unfortunately overlooked too much the influence of the nervous over the vascular system, while so intent upon the explanation of all phenomena by increased momentum of the blood.

We are ready to agree with our author, however, that any excessive action of the heart may cause a *local* accumulation of blood, where there is *local* predisposition in the vessels.

We are unable to give any analysis of Dr. Parry's Answers to the Strictures of Dr. Wilson Philip on his father's doctrines; but must refer to the volume itself, from page 160 to page 178.

The readers of the Monthly Series of this Journal will recollect, that immediately prior to Dr. Parry's ever-to-be lamented affliction, a discussion had commenced between that physician and Dr. James Johnson, one of the then Editors of the Medico-Chirurgical Journal and Review. Dr. Charles Parry has, in his notes, republished the reply of his father, and made several ingenious remarks on Dr. Johnson's rejoinder, some of which we shall here notice.

Dr. Johnson, from reflection on the subject; and from the phenomena exhibited by an apparatus intended to represent a bilocular heart, came to the conclusion that, as Dr. Parry had stated, there is neither dilatation nor contraction in the arteries, corresponding with the systole and diastole of the ventricles. But he went one step farther: he argued, that if there be no contraction of the aorta *after* each discharge of blood into it from the ventricle, there can be no progression of the blood *last received* until the succeeding systole. For, during the diastole of the ventricle, when no blood is received into the root of the aorta, how is it possible for the blood to flow along without some contraction of the parietes of the vessels, unless a vacuum were left behind? To these conclusions, Dr. Parry has opposed what he terms the "*opposite* matter of fact," which is as follows.

"In many hundred instances I have observed the current of blood. It has borne no other character than that of a rapid river, varying its degree of motion *from accidental causes alone*, and indicating in its *uniform* course no evident effect, *even from an alternating impulse*."

It may, in the first place, be observed, that Dr. Parry has drawn his "matter of fact" from a source which, in a preceding part of the work, he warns us earnestly against, as teeming with error—*microscopical observations.* In what possible way can Dr. Parry reconcile this rapid and *uniform*

*current* with the phenomena of the pulse in his father's and his own experiments? That the elder Dr. Parry had quite a different notion of the circulation appears by the following extract from his reply to Dr. Johnson.

"I consider the blood-globules, buoyant in the fluid in which they swim, as, for most purposes of reasoning, incompressible; and, in this view, they are like a series of billiard-balls in close contact with each other, so that if an impulse is made from behind, immediately the foremost ball, however distant, is moved; or, being already in motion, as in the present instance, feels the *preponderating velocity of the new impulse.*" And again, "When the left ventricle contracts, it expels its blood, as, for example, two ounces, into the aorta; but it does not add that quantity to what existed before. It only displaces an equal quantity, *causing the whole mass to advance towards the right auricle, which, at the same instant,* receives just as much of the mass as was supplied by the ventricle."

But as Dr. Parry may still oppose the "matter of fact" to trains of reasoning, we must endeavour to meet him on his own ground.

"We may derive, (says Bichat,) a very exact idea of the circulation, by examining the mesenteric arteries of a living animal through the peritonæum. *At each pulsation* they appear to be raised *at once from their origins to their extremities.* The motion is *not progressive*, and the blood does *not flow*, properly speaking, but is pushed suddenly by a *gentle shock.*"

*Anatomie Generale.* See also, *Rees's Cyclopædia, Article, Heart.*

The above are sufficient specimens of what Dr. Parry has most appropriately, though undesignedly characterized as "*opposite* matters of fact," resulting from *microscopical* observations on the circulation; and those who wish for *variety* of these, have only to consult the various experimentalists, scarcely any two of whom ever came to the same conclusion.

It is evident that Dr. Parry himself had not much confidence in the microscopical observations, when he employs such reasoning as the following, to support the doctrine of perpetual flow of blood along the aorta.

"If, (says Dr. Charles Parry,) the current of blood has not been projected *beyond the semilunar valves* into the aorta, these valves will be prevented closing; and if they do not close, the stream from the ventricle may be perpetual."

We would first ask, where can the ventricular blood be projected to, if not *beyond the valves* of the aorta? And again, how can the blood continue to be ejected from the ventricle *during* the act of its *dilatation*, especially as Dr.

Parry questions the dilatation and contraction of the auricle?

It appears, by a document which Dr. Parry has cited, indeed, that Dr. Johnson is not singular nor original in the opinion of alternate *quiescence* and motion in the arterial blood of the trunks, at least. Dr. Jurin, of Geneva, in the year 1738, observed as follows:—

"Hence, it appears, that the blood last driven out of the heart, when the systole is performed, *remains unmoved* in the arteries, &c."

A German physiologist has lately come to the same conclusion, as may be seen in the second number of the "Continental Medical Repository." The observations of Bichat, on the mesenteric arteries, certainly confirm, as far as vivisections can, the above-mentioned doctrine, strange as it may seem to minds contented with preconceived opinions. This appears to be the weakest part of Dr. Parry's work; but it is only justice to state, that he brings it forward merely as an hypothesis, naturally resorted to in a matter of such difficulty.

As a whole, the work presents a perfect model of clear and logical reasoning, founded on legitimate and well-arranged facts. We hope, for the interest of medical science, that Dr. Parry will, in imitation of his excellent and ever-to-be-remembered father, employ those talents, which Nature has bestowed, and cultivation improved, for the benefit of mankind at large, as well as his own. Let him remember the solemn, and alas! *prophetic* pledge which his father gave the profession, four years ago!

"If, however, it shall please Providence to frustrate that hope [of 'completing the work on Pathology and Therapeutics], the author has great satisfaction in leaving his materials in the hands of a person, deservedly most dear to him, of whom it may be permitted him to assert, that his talents, as well as general and professional knowledge, render him fully competent to the execution of that task."—*Preface to Elements of Pathology*, 1815.

The voice of the profession—nay, "the voice of Nature" cries aloud for the redemption of this pledge, and the execution of this *pious* task. To such calls, Dr. Parry's honour and filial affection will not permit him to turn a deaf ear.

We now come to Mr. Charles Bell's Essay. This gentleman has always felt somewhat humbled, in entering on the subject of the blood's circulation, in his functions, as a public teacher of anatomy. He has been obliged to expose to his pupils the want of just principles, and the substitution of mere authorities, in explanation of the subject.

It appears to us, that Mr. Bell, like many others, attaches a vast deal too much importance to the friction of the blood along, and attraction to the sides of the arteries:—and consequently, magnifies the force which is necessary to move the blood.

In the first place, physiologists have greatly exaggerated the *velocity* of the blood; deceived by microscopical observations, and the phenomena attending a wounded artery. Respecting the microscope, enough has been said; and therefore we shall here make some remarks on the flow of blood from an artery.

On a moderate calculation, the heart throws into the aorta about five or six pounds of blood in the minute; of which there cannot be more than a sixth part transmitted through the inguinal artery, or about fifteen ounces per minute. Let us compare this rate of healthy progression with that where an unnatural outlet is given to the circulating fluid It was once our unhappy lot to witness a wound of the inguinal artery by a very sharp instrument. About half the calibre of the vessel was divided; and we can safely aver, that in less than twenty seconds, full a gallon of blood issued from that fatal wound! Thus, in the space of one-third of a minute, *eight pounds* of blood rushed along an artery, through which *five ounces* only would have flowed, had the vessel remained entire; yet it is upon such data as these, that the prodigious velocity of the blood in healthy progression has been calculated.*

---

* "When I witness the force (says Mr. Bell) with which the blood spouts from a wounded vessel, and when I consider the amazing number and minuteness of the vessels through which the blood *is flowing at the same rate*, &c" P. 11.

A pipe bursts in our streets, and the water boils out with great force and velocity; yet the fluid was probably *quiescent* the moment before. The pressure of the reservoir, at a distance, has precisely the same effect on the motion of the water, when an outlet is made, as the action of the heart and contractility of the arteries have on the blood, when a wound is made in the vessel. In both cases, the velocity of the fluids from the artificial exits, as compared with the previous motion in the tubes, would lead the superficial observer to the most erroneous conclusions.

Bichat has justly remarked, that "the progress of physiology concerning the circulation has been much obstructed by the current notions concerning the *velocity* of the blood's motion in the arteries, of which we can form no true estimate, because the motion is not progressive, and cannot be subjected to calculation."—*Anat. Generale.* See also, *Rees's Cyclopedia; article, Heart.*

But let us examine things more accurately, and with philosophic calmness. The arteries are endued with a twofold power—vital and elastic. The former, which has been termed *organic* contractility, to distinguish it from *voluntary*, or common muscular contractility, enables it, when a free outlet is given to the blood, to completely annihilate the lumen or bore of the vessel. Thus, when a large artery, as that of the groin, is divided, both the elastic and organic powers of the *whole arterial system* press the blood from all parts, with immense force, to this new outlet, and it rushes thence, "*qua data porta*," with a velocity quite unknown in natural states of the circulation.

We are aware of the objection which Mr. Bell may make to this, in the case of divided arteries which do not bleed. We have seen many cases where a limb has been shot off, and yet no hæmorrhage succeed. But, in such instances, there was a general shock given to the whole nervous system; and the heart and *arterial system* participated therein. The vessels also being torn quite across, either contracted immediately from the violent *local* stimulus, or coagula were formed in their mouths, which arrested, *pro tempore*, the flow of blood. Yet the moment the sensorial energy was revived, the hæmorrhage returned.

Mr. Bell, surveying the vast resistance that a fluid moving with astonishing rapidity [which we trust we have shown does not exist] must encounter from the attraction and friction of its conduits, has had recourse to an hypothesis which we shall state in his own words.

"Are we prepared to admit that this universal attracton of fluids and solids is *negatived* in the vessels of a living body—that the great architect, instead of accumulating forces to overcome the vast resistance, has annihilated it, and rendered a smaller force sufficient to the end, and, at the same time, consistent with the delicate texture of our frame." P. 12.

We certainly are not prepared to deny the truth of this hypothesis, since there are several analogical phenomena, of vital powers superseding or suspending chemical and other affinities, which come in to its support. Our only objection to it is, our conviction that there is no *necessity* for its existence; in which case the Epic precept, "Nec Deus intersit, &c." should be adopted. We believe that the great Architect has wisely adapted the moving powers to the opposing resistances, without abrogating those universal laws by which, as Mr. Bell elegantly expresses it,

"All motions are begun, by which heavy bodies descend, light bodies float, rain falls, and vapours ascend."

The second part of this little work is on the *action* of arteries, and opens with a severe satire on certain modern experimental physiologists. Averse as we always are from any unnecessary cruelty towards the brute creation, and conscious that man—

> "Should view with tenderness all living forms,"

Yet we cannot participate in Mr. Bell's excessive sensibility on this occasion. The death of a few rabbits, for the purposes of useful science, is certainly not much calculated to call forth a rhapsodical anathema against the sin of "*torturing* living animals," while millions of them are weekly slaughtered to sate the appetites of pampered epicures. We have every reason to believe that strong sense far predominates over morbid sensibility in the profession; and that small will be the number of those who shall join in the vulgar cry of cruelty against a Parry and a Philip, for their highly meritorious labours in the advancement of medical science, even when done at the expence of pain or death to the subjects of their experiments.

Mr. Bell, though an advocate for the doctrine of *alternate* dilatation and contraction in the arteries, corresponding with the pulse, gives up the attempt at proving it by demonstration. These alterations of the capacity of vessels, he acknowledges, are *imperceptible to the eye or touch.* We shall not therefore waste time in combating such shadowy substances as elude our senses; but claim from Mr. Bell the reasonable admission that the *effects* of these implied alternate dilatations and contractions must be proportionate to their *causes*; that is, *incognizable also by the senses.* This granted, the old logical conclusion—"de non apparentibus et non existentibus, eadam est ratio," will be easily drawn.

> "To suppose, says Mr. Bell, that the heart is the only engine, or even that it is the *principal* cause of the motion of the blood, must leave us in perfect astonishment, when we see it ossified in its substance, or encumbered with a tumour which surrounds it wholly, and adheres to it on all sides."

Our author then quotes the case of Margaret Henderson, from Allan Burns, where the ventricles, except about a cubic inch, "were ossified and firm as the skull."

This passage may certainly excite the astonishment of those who take it as here stated, without inquiring farther; for it appears intended to convey the idea that Margaret Henderson got through the world with her heart of bone, as well as those whose hearts are composed of more tender materials. But a perusal of the case will shew the dreadful state to which this poor woman was reduced—"compelled to continue constantly in a semi-erect position, a recumbent posture never failing to induce a paroxysm of breathlessness." *Burns, Page* 130.

But what are the remarks of Mr. Burns, on this case, who himself makes the arteries almost omnipotent in the circulation?

"When the ventricles, says he, have their power annihilated, the auricles are necessitated, in part, to perform both their own functions and also that of the ventricles, and to accomplish this, it is required that their muscularity be also augmented. *When the ventricles are ossified, we uniformly find the flesh of the auricles firmer and stronger than usual.*" Burns, Page 129.

Such was the case in Margaret Henderson, and yet the *vicarious* function of the auricles was unequal to the task of supporting life! The reader therefore will judge of the strength of Mr. Bell's argument for the independent power of the arteries, when grounded on such facts as the above.*

It is now fair for us to turn Mr. Bell's argument against himself. Let us reverse the picture. Numerous examples are on record of the whole tree of *arteries* being ossified, and yet the circulation going on well, and the patients (if patients they might be called) arriving at ages far above 100 years. The history of old Bayles, recorded in the Philosophical Transactions, who lived to the age of 130, with all the great arteries of the body ossified, is sufficiently in point. A remarkable instance, also, has lately been published, in the Medico-Chirurgical Transactions, of a very old man, who apparently laboured under no other disease than that of extreme old age, and in whom the arteries were found ossified, even to the wrists and ankles.

In this part of the work Mr. Bell has wasted a great deal of time and argument in combating a shadow, or

---

* It is worthy of remark, that in Margaret Henderson's case, notwithstanding the organic derangement of the heart, the pulse preserved a considerable degree of regularity. Now that the *pulse* is caused by the action of the *heart*, and not by that of the *arteries*, we have the authority of Mr. Charles Bell himself, in his System of Dissections. "It is not the degree of dilatation, says he, which we feel in the *pulse*, but the shock given to the column of blood by the *action of the ventricles.*" The phenomenon of the pulse, therefore, in Margaret Henderson, proves the *action of the heart*, and not that of the arteries, even by our author's own confession.

rather a phantom which no where exists. Against those who advocate the doctrine of no *alternate* dilatation and contraction in arteries, corresponding with the systole and diastole of the ventricles, their opponents are perpetually crying out, as though they compared the aforesaid vessels to "inert tubes," devoid of any other power than mechanical elasticity! Such accusations have any thing but truth for their foundation. Bichat, in France, and Parry, in England, (to say nothing of a host of other eminent men) have clearly demonstrated the *vital* as well as the elastic power of arteries. Bichat terms it *organic contractility*; Parry, *tonicity*, or vital force. These two powers are universally and unanimously granted to the said vessels, here and on the continent. How trifling is it then to bring forward a gorgeous force and pomp of argument against a non-entity.

True it is, that on both sides of the channel, this vital power of arteries has been recognized as differing materially from common voluntary, or *muscular* contractility. Bichat, in particular, has most luminously pointed out this difference, in his General Anatomy; and the English reader may satisfy himself of this, by turning to the article "*Heart*," in Rees's Cyclopœdia.

But while we deny to arteries any share in the *progression* of the blood, at each systole of the heart, under ordinary states of the circulation, we consider that their vital and elastic powers are almost constantly employed in changing the relative volume of blood in different parts of the body; either in obedience to various external and internal agents, or to adjust the balance to the exigences of the system. Thus if cold be applied to the surface of the body, the vessels will contract, by virtue of their *vital* or tonic power, and the blood is thrown on the more interior vessels. Presently these react, by the same power, and again restore the balance of the circulation. If, on the other hand, an inordinate share of nervous power be directed or solicited to any part of the system, for the necessary purposes of the constitution, the vital power of the arteries puts *them* in immediate co-operation with the nerves, for effecting whatever function is required, as secretion, utero-gestation, growth of parts, &c. &c. These are some of the great and important ends for which, we conceive, the vital and mechanical properties were conferred on the arteries. They are more dignified functions than our opponents allot them, although they accuse us of degrading them to the office of "inert tubes."

But our limits are already exceeded, and the article must come to an immediate close; though we could bring forward much on this subject that might prove both useful and interesting. We presume not to sit in *judgment* on the work of such a man as Mr. Charles Bell. We simply oppose fact to fact, and argument to argument; neither seeking importance nor fearing derogation from the anonymous veil that surrounds us. We are, ourselves, at the bar of the public tribunal, where we well know that nothing can avail us but the independence of our sentiments—the justice of our cause—and the force of truth. By these we are *guided;* and by these we hope to be *judged.* Whenever these cease to be cherished by that public whom we serve —" Othello's occupation's gone!"

